# Sex differences in metabolic phenotype and hypothalamic inflammation in the 3xTg-AD mouse model of Alzheimer’s disease

**DOI:** 10.1186/s13293-023-00536-5

**Published:** 2023-08-09

**Authors:** Lisa S. Robison, Olivia J. Gannon, Abigail E. Salinero, Charly Abi-Ghanem, Richard D. Kelly, David A. Riccio, Febronia M. Mansour, Kristen L. Zuloaga

**Affiliations:** 1 Department of Neuroscience and Experimental Therapeutics, Albany Medical College, 47 New Scotland Avenue, Albany, NY 12208 USA; 2 Department of Psychology and Neuroscience, Nova Southeastern University, 3300 S. University Drive, Fort Lauderdale, FL 33328 USA

**Keywords:** Sex, Gender, Alzheimer’s disease, Inflammation, Hypothalamus, Metabolic

## Abstract

**Background:**

Alzheimer’s disease (AD) is notably associated with cognitive decline resulting from impaired function of hippocampal and cortical areas; however, several other domains and corresponding brain regions are affected. One such brain region is the hypothalamus, shown to atrophy and develop amyloid and tau pathology in AD patients. The hypothalamus controls several functions necessary for survival, including energy and glucose homeostasis. Changes in appetite and body weight are common in AD, often seen several years prior to the onset of cognitive symptoms. Therefore, altered metabolic processes may serve as a biomarker for AD, as well as a target for treatment, considering they are likely both a result of pathological changes and contributor to disease progression. Previously, we reported sexually dimorphic metabolic disturbances in ~ 7-month-old 3xTg-AD mice, accompanied by differences in systemic and hypothalamic inflammation.

**Methods:**

In the current study, we investigated metabolic outcomes and hypothalamic inflammation in 3xTg-AD males and females at 3, 6, 9, and 12 months of age to determine when these sex differences emerge.

**Results:**

In agreement with our previous study, AD males displayed less weight gain and adiposity, as well as reduced blood glucose levels following a glucose challenge, compared to females. These trends were apparent by 6–9 months of age, coinciding with increased expression of inflammatory markers (Iba1, GFAP, TNF-α, and IL-1β) in the hypothalamus of AD males.

**Conclusions:**

These findings provide additional evidence for sex-dependent effects of AD pathology on energy and glucose homeostasis, which may be linked to hypothalamic inflammation.

## Plain language summary

Alzheimer’s disease (AD), often associated with memory loss, can also affect other parts of the brain and body, resulting in several other symptoms. Changes in appetite and body weight are commonly seen in people with AD, often before they start showing signs of memory loss. These metabolism-related changes are likely due in part to AD affecting a part of the brain called the hypothalamus, which controls important functions like energy balance (calories in vs. calories out) and blood sugar levels. This study aimed to examine whether changes in metabolism and the hypothalamus could serve as early signs of AD, and even help in treating the disease. We also wanted to see if these changes were influenced by biological sex, as two-thirds of AD patients are women, and our previous studies showed many differences between males and females. In this study, we observed male and female mice at different ages to see when these changes began to appear. We found that male AD mice gained less weight, had less body fat, and had better blood sugar control, compared to female AD mice. These differences became noticeable at the same age that we noticed signs of increased inflammation in the hypothalamus of male mice. These findings suggest that AD affects males and females differently, particularly in terms of energy balance and blood sugar control, and this might be related to inflammation in the hypothalamus. This research could provide valuable insights into understanding, diagnosing, and treating Alzheimer’s disease.

## Highlights

- Alzheimer’s disease (AD) not only affects memory, but also impacts the hypothalamus, a part of the brain controlling energy balance and glucose homeostasis.
- Changes in appetite and weight are common in AD and can precede memory loss symptoms. These could potentially serve as AD biomarkers or treatment targets.
- This study found sex-specific differences in metabolic outcomes and hypothalamic inflammation in 3xTg-AD mice.
- Male 3xTg-AD mice had less weight gain and adiposity (subcutaneous and visceral), and improved glucose tolerance, compared to females starting from 6 to 9 months of age.
- These changes in male mice coincided with increased expression of pro-inflammatory cytokines in the hypothalamus, suggesting a sex-dependent effect of AD.

## Background

Alzheimer’s disease (AD) is the most common contributor to dementia, a disabling and deadly disease that has become a major public health burden, affecting more than 55 million people worldwide. It is estimated that global prevalence will increase to 78 million people in 2030 and 139 million in 2050 [1]. Beta-amyloid (Aβ) plaques and neurofibrillary tangles have long been considered hallmarks of the disease, associated with neuroinflammation and neurodegeneration [2]. Attention has primarily focused on the cognitive symptoms of AD (e.g., memory impairment, confusion, shortened attention span) and associated brain regions like the hippocampus and other cortical areas are the most widely studied; however, this disease can also result in several non-cognitive symptoms and pathology in widespread brain areas [3]. These non-cognitive symptoms of AD may accelerate disease progression, contribute to poor quality of life, and may even be life-threatening. Additionally, investigation of these other domains and brain regions, some of which are believed to be affected a decade or more before the onset of cognitive decline, may expose new biomarkers or targets for treatment.

Metabolic disturbances comprise one such group of non-cognitive symptoms often present in AD patients. Disturbances in feeding behavior and metabolic function, as well as changes in body weight and composition, are commonly seen in both AD patients and mouse models [3–18]. Late-life weight loss and low BMI are associated with an increased risk of dementia, greater amyloid burden, faster progression of the disease, and increased morbidity and mortality [8–13]. The association between high BMI/metabolic disease (obesity, type 2 diabetes) and dementia risk appears to vary by age: higher BMI in late life is protective [19], while obesity and type 2 diabetes in mid-life are associated with increased risk of AD [14, 15]. Longitudinal studies suggest that instability of BMI in either direction is predictive of outcomes, such that both weight loss or weight gain (e.g., > 5% change) is associated with greater risk for cognitive decline or progression to AD/dementia [16–18].

Further investigation of these relationships between metabolic alterations and dementia risk/progression is important for several reasons. Unfortunately, most medications currently approved for AD, such acetylcholinesterase inhibitors and NMDA receptor antagonists, have shown limited benefit, only temporarily relieving symptoms without modifying the disease process. The recently approved Aβ-directed monoclonal antibody, aducanumab, facilitates amyloid removal but has questionable benefits for cognition and carries substantial risk for amyloid-related imaging abnormality (ARIA), including brain swelling and/or bleeding [20]. A plethora of other disease-modifying drugs have failed in clinical trials, as they were not capable of halting or reversing the progression of the disease, likely because treatment begins too late in the disease process. Diagnosis of dementia occurs, and treatment commences after the onset of cognitive symptoms; however, AD pathology can begin developing ~ 20 years prior. Changes in body weight can be seen a decade or more prior to cognitive decline [21–23]. This highlights the possibility of using metabolic markers as early biomarkers of the disease to identify and intervene for at-risk individuals.

Altered metabolic function in AD may be a result of pathological changes in the hypothalamus, which is the master regulator of homeostatic functions via modulation of endocrine and autonomic function and regulation of circadian rhythms. The hypothalamus plays a role in energy and glucose homeostasis, balancing food consumption with energy expenditure, and regulating the release of glucose from the liver. Several studies have identified early and significant neuropathological changes in the hypothalamus of AD patients, including an accumulation of amyloid and tau, atrophy, reduced glucose metabolism, and trends of hypoperfusion [3, 24–28]. A recently reported retrospective longitudinal study found that neuropathological changes in the hypothalamus were similar in magnitude to changes seen in the hippocampus, and that hypothalamic atrophy was associated with AD biomarkers (CSF levels of Aβ42, tau, and phosphorylated-tau) [29]. Hypothalamic abnormalities and impaired function have also been shown in mouse models of AD [6, 30–33]. Of note, sex differences in hypothalamic changes and metabolic outcomes have been noted in dementia patients and AD mouse models [25, 34]. Investigation of sex differences is important for nearly all diseases, as sex can influence the risk and progression of the disease, manifestation of symptoms, and responses to treatment [35, 36]. However, this may be particularly crucial for conditions with a clear sex/gender bias, such as AD, for which women have approximately double the risk compared to men [37, 38].

The hypothalamus is not only a target of AD pathology, but additionally, metabolic and hypothalamic dysfunction have been theorized to be key drivers of neurodegenerative processes and dementia symptomology [3]. For example, glucose, leptin, and insulin not only play a role in maintaining metabolic homeostasis, but are also vital to maintaining cognitive function and influence amyloid processing, tau, and inflammation. AD has been argued to be “Type 3 diabetes” [39], since the brains of AD patients and rodent models often display impairments in brain insulin responsiveness, glucose utilization, and energy metabolism [40–45], while treatments aimed to ameliorate these impairments can improve cognitive function [45–52]. Taken together, these findings suggest that metabolic disturbances associated with AD may represent novel targets for treatment.

Previously, we reported sexually dimorphic metabolic disturbances and hypothalamic inflammation in 7- to 8-month-old 3xTg-AD mice (a mouse model of AD) [34]. Interestingly, these mice appeared to represent distinct metabolic phenotypes, similar to the bimodal distribution of weight changes (i.e., significant weight loss and weight gain) reported in AD patients and associated with increased risk for cognitive decline [16–18]. In our previous study, control diet-fed AD males displayed attenuated weight gain and adiposity compared to wild-type (WT) controls and AD females, as well as reduced blood glucose levels following a glucose challenge, accompanied by increased expression of pro-inflammatory markers (Iba1, GFAP, TNF-α, and IL-1β) in the hypothalamus. Additionally, AD females particularly on a high-fat diet exhibited disproportionately increased weight gain and adiposity, as well as glucose intolerance, associated with astrogliosis in several nuclei of the mediobasal hypothalamus that regulate energy homeostasis (arcuate nucleus, dorsomedial nucleus, ventromedial nucleus). In the current study, we investigated metabolic outcomes and hypothalamic inflammation in 3xTg-AD males and females at 3, 6, 9, and 12 months to determine when these sex-specific metabolic phenotypes emerge and how they change over the course of adulthood.

## Method s

### Animals

This study was conducted in accordance with the National Institutes of Health guidelines for the care and use of animals in research, and protocols were approved by the Institutional Animal Care and Use Committee at Albany Medical College, Albany, NY, USA. Male and female 3xTg-AD breeder pairs (#34830-JAX) were obtained from Jackson Laboratories (Bar Harbor, Maine) and were used to generate male and female 3xTg-AD mice for this experiment. These 3xTg-AD mice are on a C57BL/6;129X1/SvJ;129S1/Sv genetic background and exhibit three human mutant genes that result in familial AD, including APPSwe, tauP301L, and Psen1tm1Mpm [53]. By 3–4 months, intracellular Aβ deposition is apparent. Extracellular Aβ deposition, impaired synaptic transmission and LTP is observed by 6 months, and hippocampal deposits of hyperphosphorylated tau at 12–15 months [53, 54].

Mice were group-housed following weaning, usually with same-sex littermates with 3–5 mice per cage. Mice were generally left undisturbed in the animal facility, except for weekly cage change and handling. Male and females 3xTg-AD mice were aged out to 3, 6, 9, and 12 months of age (3 months male *n* = 6, 3 months female *n* = 10, 6 months male *n* = 10, 6 months female *n* = 10, 9 months male *n* = 10, 9 months female *n* = 10, 12 months male *n* = 7, 12 months female *n* = 9). About 1 week before mice reached their assigned terminal age point, they underwent a glucose tolerance test (GTT) to assess diabetic status. Mice were euthanized under deep anesthesia (~ 4% isoflurane), and blood (cardiac puncture) and tissues were collected. Wet weights were taken of heart and fat pads (visceral and subcutaneous).

### Glucose tolerance test

Mice were fasted overnight, and baseline glucose levels were measured by standard commercial glucometer (Breeze 2, Bayer, Tarrytown, NY). Each mouse received an i.p. injection of 20% glucose at 10 μL/g of body weight, and blood glucose levels were then re-measured at 15, 30, 60, 90, and 120 min. Blood was collected via tail snip (< 1 mm) prior to glucose injection, and at each subsequent time point, the clot was gently removed to collect subsequent samples. Area under the curve (AUC) was calculated for each mouse. It should be noted that repeated blood sampling following tail snip procedure in mice can elicit a stress response (hypercortisolemia) [55]; therefore, it is possible that hypothalamic gene expression levels were influenced by this procedure, which occurred one week prior to tissue collection.

### Quantitative polymerase chain reaction (qPCR)

Brains were rapidly removed, and the hypothalamus was microdissected while emerged in PBS over dry ice. Microdissections were then stored at − 80 °C until total RNA isolation was performed using the TRIZOL method. cDNA was prepared using 1 μg RNA and the High-Capacity cDNA Reverse Transcription Kit (Applied Biosystems, Catalog number: 4368814). The qPCR experiments were performed using TaqMan Gene Expression Master Mix (Applied Biosystems, Catalog number: 4369016) in the presence of Taqman Assays with primer/probes for Iba1 (Mm00479862_g1), GFAP (Mm01253033_m1), IL-1β (Mm00434228_m1), TNF-α (Mm00443258_m1), IL-6 (Mm00446190_m1), and Ikbkb (Mm01222247_m1), and human APP (Hs00169098_m1) as target genes, and β-actin (Mm02619580_g1), RPL13A (Mm01612986_gH), and RPS17 (Mm01314921_g1) as housekeeping genes. Water (negative control), as well as a reference/positive control (homogenate sample of hypothalamus collected from five 3-month-old WT mice), were run alongside samples. Data were plotted as relative normalized expression compared to WT control sample. Graphing and statistical analyses of qPCR data were performed using CFX Maestro Software (Bio-Rad Laboratories, Inc., Hercules, CA, USA) with significance set at *p* < 0.05.

### Statistical analysis

All data are expressed as mean ± SEM. Pearson correlations were run to determine whether there were sex-specific associations between metabolic outcomes and hypothalamic gene expression. A three-way repeated measures ANOVA (between-subjects measures: sex and age; within-subject measure: time) was used for analysis of blood glucose levels over time during the GTT test. Analyses of all other measures were performed using two-way ANOVA (between-subjects measures: sex and age) followed by post hoc tests (Holm–Sidak method) when appropriate. Grubbs test was performed with alpha = 0.05 to remove statistical outliers. Outliers were removed for the following: one 3-month-old male each for hypothalamic expression of *TNF-α* and *IL-6*, and one 3-month-old female each for hypothalamic expression of *TNF-α* and *Ikbkb*. Statistical significance was set at *p* < 0.05, and statistical analyses were performed using GraphPad Prism v9 software.

## Results

### Metabolic outcomes

#### Body mass

**Fig. 1:**
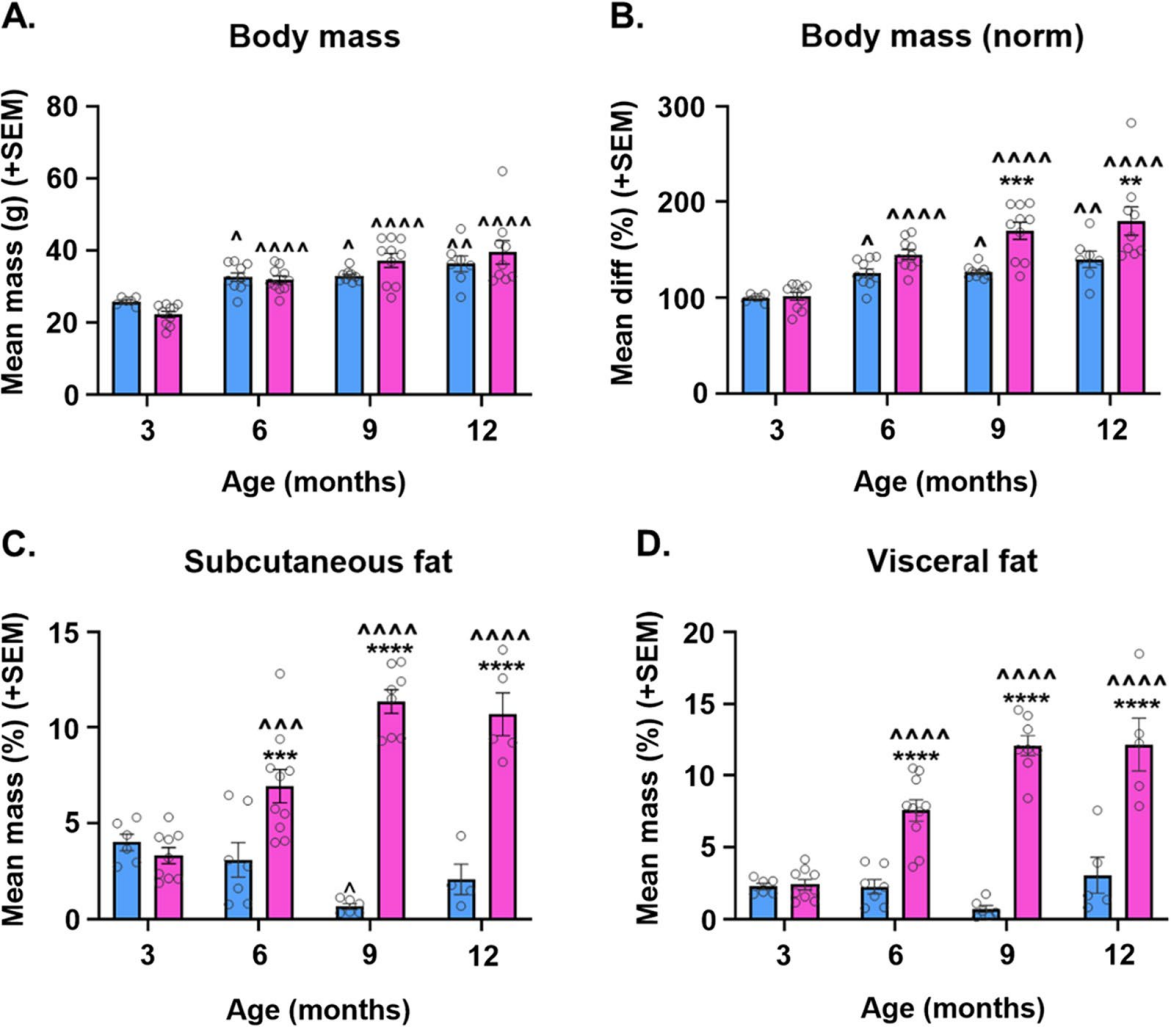
Sex and age interact to influence body mass and adiposity in 3xTg-AD mice. **A** Raw body mass in male and female 3xTg-AD mice at 3, 6, 9, and 12 months of age. *n* = 6–10/group. **B** Body mass normalized to same-sex mice at 3 months of age in male and female 3xTg-AD mice *n* = 6–10/group. **C** Subcutaneous adiposity, as calculated by percent body mass, in male and female 3xTg-AD mice at 3, 6, 9, and 12 months of age. *n* = 4–10/group. **D** Visceral adiposity, as calculated by percent body mass, in male and female 3xTg-AD mice at 3, 6, 9, and 12 months of age. *n* = 5–10/group. **p < 0.01 vs. opposite sex of same age, ***p < 0.001 vs. opposite sex of same age, ****p < 0.0001 vs. opposite sex of same age, ^ = *p* < 0.05 vs. same-sex 3 month; ^^ = *p* < 0.01 vs. same-sex 3 month; ^^^ = *p* < 0.001 vs. same-sex 3 month; ^^^^ = *p* < 0.0001 vs. same-sex 3 month. Data are presented as mean ± SEM

Body mass at the end of the study is presented as raw mass (Fig. 1A) and mass normalized to same-sex mice at 3 months of age (Fig. 1B). The latter was calculated to account for normal sex differences in body mass (males > females). As expected, body mass was greater at older ages (main effect of age; *p* < 0.0001 for both raw and normalized body mass). For normalized body mass, there was a main effect of sex (*p* < 0.0001; F > M) and sex x age interaction (*p* = 0.0362). Pairwise comparisons revealed that there was an increase in body mass in both males and females from 3 months of age to all later age groups. However, in 9- and 12-month-old mice, females exhibited greater normalized body mass than males.

#### Fat mass

Subcutaneous (Fig. 1C) and visceral (Fig. 1D) fat pads were collected, weighed, and normalized fat mass was calculated as % of body weight. Females overall had greater body fat % compared to males (main effect of sex; subcutaneous and visceral *p* < 0.0001). Both subcutaneous and visceral fat tended to be greater at later age points (main effect of age; subcutaneous *p* = 0.0041, visceral *p* < 0.0001); however, this was clearly driven by the sex × age interactions (*p* < 0.0001 for both subcutaneous and visceral fat). In females, both subcutaneous and visceral fat were increased at 6, 9, and 12 months compared to 3-month-old mice (*p* < 0.001 for all); however, in males, fat mass was not increased at later ages. In fact, subcutaneous fat mass was decreased in 9-month males compared to 3-month males (*p* = 0.012). Additionally, direct comparison of the sexes revealed that females had more visceral and subcutaneous fat than males at 6, 9, and 12 months (*p* < 0.001 for all).

#### Glucose tolerance

**Fig. 2:**
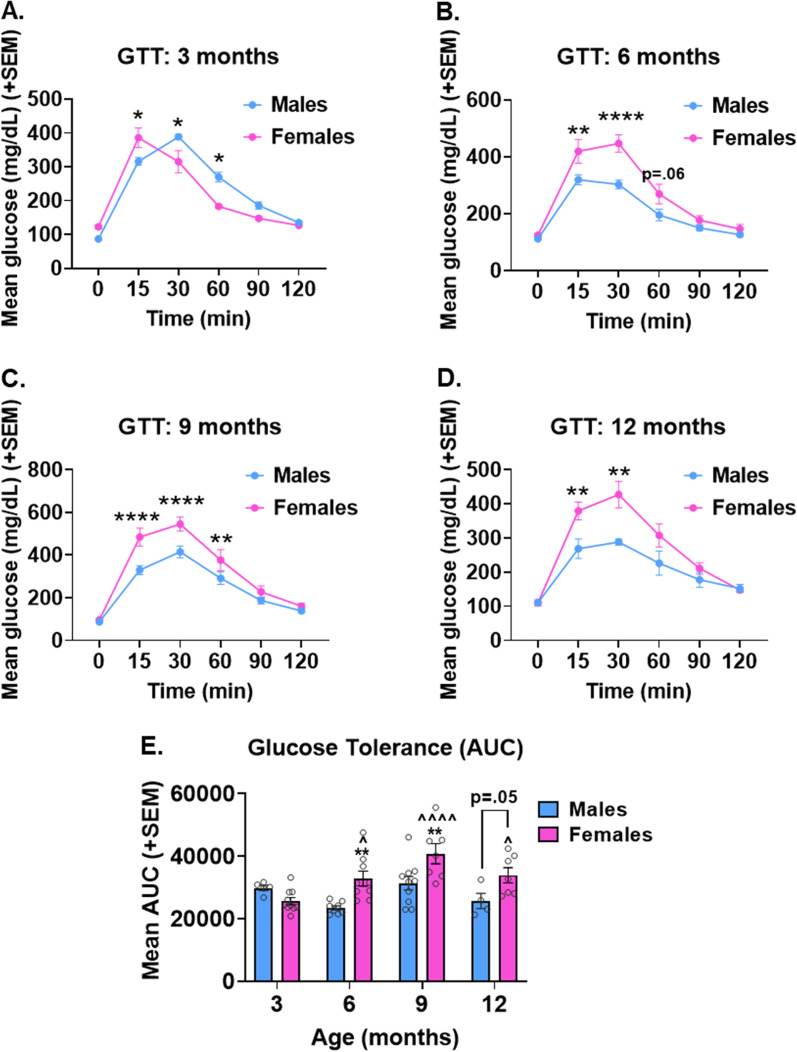
Sex and age interact to influence glucose intolerance in 3xTg-AD mice. **A–D** Blood glucose levels were measured following overnight fasting (*t* = 0), then mice were subjected to a glucose tolerance test (GTT). Mice were injected with glucose challenge and blood glucose levels were measured at 15, 30, 60, 90, and 120 min post-injection. Graphs show results of the glucose tolerance test in 3xTg-AD male and female mice at **A** 3, **B** 6, **C** 9, and **D** 12 months of age. **E** Area under the curve for GTT testing was computed as a measure of total glucose exposure. *n* = 4–10/group. *p < .05 vs. opposite sex of same age, **p < 0.01 vs. opposite sex of same age, ****p < 0.0001 vs. opposite sex of same age, ^ = *p* < 0.05 vs. same-sex 3 month; ^^^^ = *p* < 0.0001 vs. same-sex 3 month. GTT = glucose tolerance test, AUC = area under the curve, mi*n* = minutes. Data are presented as mean ± SEM

**Fig. 3:**
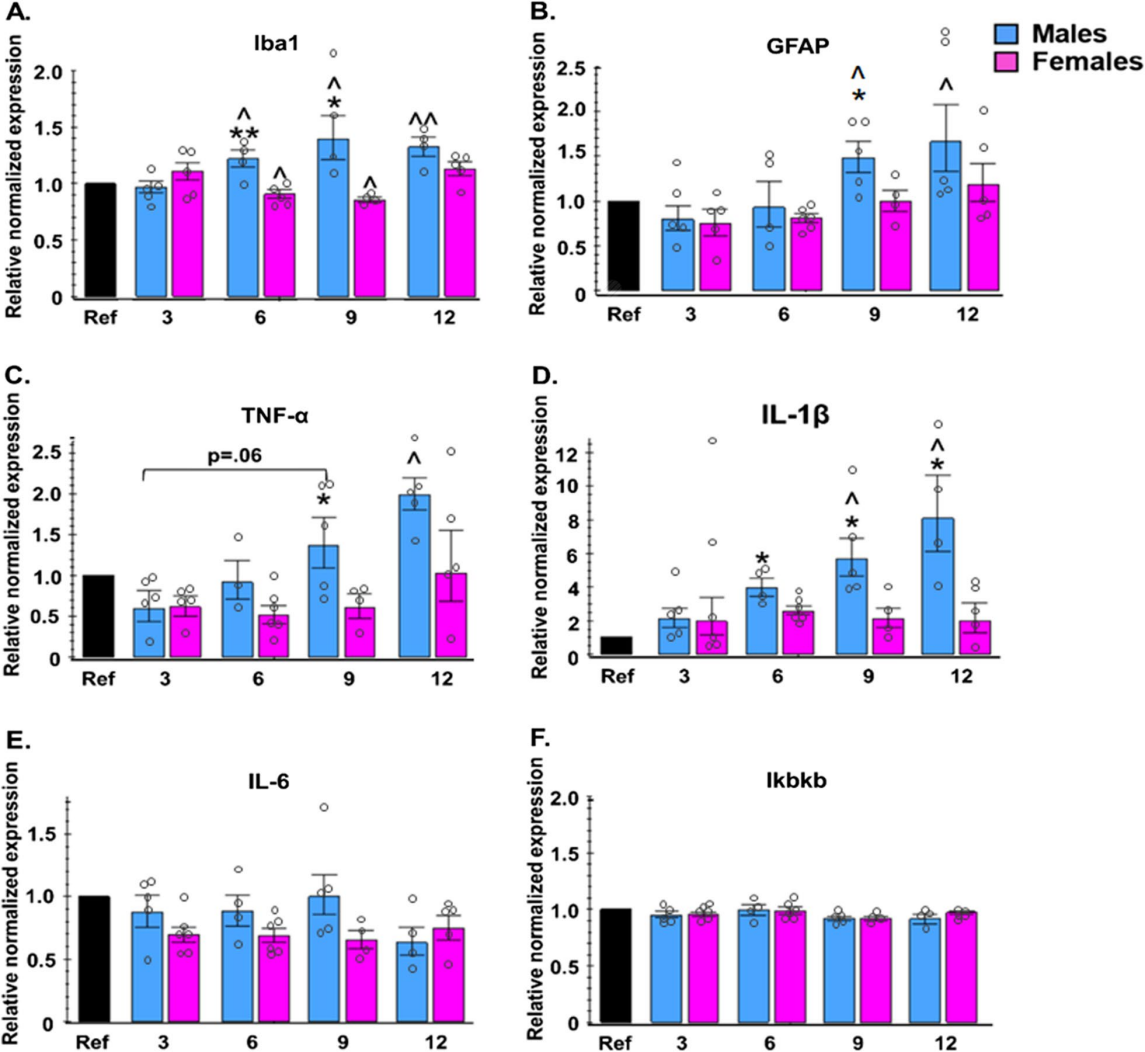
Sex and age interact to influence hypothalamic expression of inflammation-related genes in 3xTg-AD mice. Gene expression levels in homogenate of the whole hypothalamus related to inflammation in male and female 3xTg-AD mice at 3, 6, 9, and 12 months of age. Hypothalamus was collected and assayed for **A** Iba1, **B** GFAP, **C** TNF-α, **D** IL-1β, **E** IL-6, and **F** Ikbkb. Relative normalized expression levels are shown as fold-change compared to a reference sample (Ref), which was a homogenate of hypothalamic samples collected from five 3-month-old wild-type mice. *n* = 3–6/group. *p < 0.05 vs. opposite sex of same age; **p < 0.01 vs. opposite sex of same age; ^ = *p* < 0.05 vs. same-sex 3 month; ^^ = *p* < 0.01 vs. same-sex 3 month. Data are presented as mean ± SEM. Iba1 = ionized calcium-binding adaptor molecule 1, GFA*P* = glial fibrillary acidic protein, TNF-α = tumor necrosis factor alpha, IL-1β = interleukin-1 beta; IL6 = interleukin-6, Ikbkb = inhibitor of nuclear factor kappa-B kinase subunit beta

Glucose tolerance testing was performed to assess diabetic status (Fig. 2). Blood glucose levels were measured at baseline following overnight fasting, then 15, 30, 60, 90, and 120 min following a glucose challenge injection (Fig. 2A–D). Area under the curve (AUC) was calculated for each mouse as a measure of cumulative glucose exposure (Fig. 2E). In mice of all ages, blood glucose levels varied over time, as levels rose following glucose administration and fell later as glucose was cleared from the blood stream (main effect time, *p* < 0.0001 for all ages). Overall, females had impaired glucose tolerance compared to males, displayed as higher blood glucose levels. This was exhibited as a main effect of sex at 6, 9, and 12 months of age for blood glucose levels over time (*p* < 0.0001 for all; F > M), as well as a main effect of sex for GTT AUC (*p* = 0.0006; F > M). Analysis of blood glucose levels over time revealed a significant sex × time interaction at all ages (*p* < 0.01 for all). Blood glucose levels tended to be greater from 15 to 60 min post-injection of glucose in females compared to males; however, fasting blood glucose levels did not differ between sexes. Additionally, glucose appeared to be cleared with similar efficiency in males and females by the end of the GTT test (no sex difference at 90 or 120 min post-injection at any age). Analysis of AUC for the GTT test revealed a significant sex × age interaction (*p* = 0.0066). Pairwise comparisons revealed that while AUC for blood glucose remained stable across age groups in males, AUC was increased at older ages compared to 3-month mice within females (*p* < 0.05 vs. 6, 9, and 12 months). Additionally, females had greater AUC at 6, 9, and 12 months compared to males (*p* ≤ 0.05) (Fig. 3).

### Hypothalamic gene expression

RT-qPCR was performed on hypothalamus samples to determine the gene expression levels of markers for microglia (*Iba1*), astrocytes (*GFAP*), and pro-inflammatory cytokines and kinases (*TNF-α, IL-1β, IL-6,* and *Ikbkb*). Gene expression levels of human APP in the hypothalamus were also assessed; however, there were no sex differences in APP expression at any age (p > 0.10 for all; data not shown).

In males, the expression of Iba1 was increased compared to 3-month mice in 6- (*p* = 0.030), 9- (*p* = 0.039) and 12- (*p* = 0.007) month-old mice. The opposite trend was seen in females, such that *Iba1* levels were decreased in 6 (*p* = 0.031) and 9 (*p* = 0.019) month compared to 3-month-old mice. Comparing the sexes directly, males had significantly higher expression of *Iba1* compared to age-matched females at 6 (*p* = 0.003) and 9 (*p* = 0.017) months of age.

In males, the expression of *GFAP* was increased compared to 3-month mice in 9 (*p* = 0.018) and 12 (*p* = 0.033) month mice, while in females, *GFAP* levels were not significantly different over time. Additionally, males had significantly higher expression of *GFAP* compared to age-matched females at 9 months of age (*p* = 0.049).

In males, the expression of *TNF-α* was increased compared to 3-month-old mice in 9 (*p* = 0.060) and 12 (*p* = 0.005) month mice, while in females, *TNF-α* levels remained steady over time. Additionally, males had significantly higher expression of *TNF-α* compared to age-matched females at 9 months of age (*p* = 0.042).

In males, the expression of *IL-1β* was increased compared to 3-month-old mice in 9- (*p* = 0.018) and 12- (*p* = 0.011) month-old mice, while in females, *IL-1β* levels remained steady over time. Additionally, males had significantly higher expression of *IL-1β* compared to age-matched females at 6 (*p* = 0.036), 9 (*p* = 0.018) and 12 months of age (*p* = 0.037).

The gene expression levels of *IL-6* and *Ikbkb* were stable over time and did not significantly differ between sexes at any age point. In 9-month-old mice, there was a trend of females having lower *IL-6* expression compared to males (*p* = 0.070).

### Correlations

Correlations were run to determine whether there were sex-specific associations between metabolic outcomes and hypothalamic gene expression for inflammatory markers. In males, significant positive relationships were observed between body weight and Iba1 [*r*(17) = 0.4774, *p* = 0.0451], GFAP [*r*(17) = 0.5913, *p* = 0.0098], IL-1β [*r*(16) = 0.6833, *p* = 0.0025], TNF-α [*r*(16) = 0.5010, *p* = 0.0405]. Additionally, there was a positive association between visceral fat (%) and GFAP expression [*r*(12) = 0.561, *p* = 0.046] in males. In females, GFAP expression was positively correlated with body weight [*r*(18) = 0.636, *p* = 0.003], visceral fat (%) [*r*(18) = 0.580, *p* = 0.009], and subcutaneous fat (%) [*r*(18) = 0.609, *p* = 0.006].

## Discussion

In the current cross-sectional aging study, we investigated sex differences in metabolic outcomes and hypothalamic expression of inflammation-related genes in the 3xTg-AD mouse model of AD from young to middle adulthood. Male and female 3xTg-AD mice were essentially metabolically indistinguishable at 3 months of age; however, at 6 months of age, AD males displayed reduced weight gain and adiposity, as well as reduced blood glucose levels following a glucose challenge, compared to females. These findings suggest that metabolic disturbances progress with disease status. Additionally, we note increased expression of inflammatory markers (Iba1, GFAP, TNF-α, and IL-1β) in the hypothalamus of AD males starting at 6–9 months of age, with no sex differences in APP expression at any age analyzed. These findings provide additional evidence for sex-dependent effects of AD pathology on altered metabolism, which may be linked to hypothalamic inflammation.

Recently, we reported that at ~ 7 months of age, male and female 3xTg-AD mice exhibit distinct metabolic phenotypes. Compared to WT mice and AD females, male 3xTg-AD mice displayed decreased weight gain, reduced fat mass, and better glucose tolerance (7). This was in line with attenuated body mass reported previously in other AD rodent models [5, 7, 56], as well as clinical studies reporting that weight loss/low BMI are associated with dementia [8–13]. In fact, one study in male 3xTg-AD mice determined that body mass was reduced compared to WT controls at ~ 7.5 months of age [57]. Our previous study also reported that female 3xTg-AD mice displayed metabolic disturbances opposite that of 3xTg-AD males (increased body and fat mass, impaired glucose tolerance) [6]. The current study narrows down the time frame during which these sex-specific metabolic phenotypes appear, with 3xTg-AD males and females diverging on weight gain by 9 months of age, and adiposity and glucose tolerance by 6 months of age.

Our previously reported findings of an energy deficit state in 7- to 8-month-old 3xTg-AD males (approximate age at which we noted sex differences in metabolic outcomes and hypothalamic inflammatory markers here) do not appear to be due to reduced energy consumption or increased locomotor behavior [6]. In fact, hypothalamic expression of orexigenic peptides (NPY, trend for AgRP) was increased in 3xTg-AD males, likely in an attempt to drive compensatory increases in caloric consumption [6]. In line with these findings, plasma levels of the orexigenic gut hormone ghrelin were increased, while plasma levels of leptin were nearly undetectable likely due to low adiposity [6].

Metabolic disturbances (i.e., an energy deficit state) in 3xTg-AD may be attributable to increased resting metabolic rate, which has been demonstrated in other AD mouse models [56], as well as MCI and AD patients [58]. While resting metabolic rate was not assessed in our prior or current study, a previous study in 3xTg-AD male mice reported hypermetabolism (increased oxygen consumption and carbon dioxide production) at 12 months of age, but not 2 months of age; resting metabolic rate was not measured between these two age points [57]. One possible explanation is that systemic and hypothalamic inflammation exhibited by these 3xTg-AD males may contribute to this energy deficit state [6, 59]. Several nuclei of the hypothalamus play a role in energy and glucose homeostasis, balancing food consumption with energy expenditure, and regulating the release of glucose from the liver [60]. In the current study, we report that males exhibit increased hypothalamic expression of inflammation-related genes compared to females, including Iba1 (microglia/macrophage marker), GFAP (astrocyte marker), and pro-inflammatory cytokines TNF-α and IL-1β, by 6–9 months of age, coinciding with the same timeframe during which we observed sex-based divergence of metabolic outcome measures. This adds to a growing literature highlighting hypothalamic disturbances, including increased inflammation, in AD patients and rodent models [3, 4, 24–27, 30, 33, 61].

Correlational analyses were also run to determine whether there were sex-specific associations between metabolic outcomes and hypothalamic inflammatory gene expression. In males, significant positive relationships were observed between body weight and Iba1, GFAP, IL-1β, and TNF-α, as well as between visceral fat (%) and GFAP expression. In females, GFAP expression was positively correlated with body weight, visceral fat (%), and subcutaneous fat. However, since most metabolic outcomes like body weight and adiposity tended to increase with age, it is difficult to determine whether these relationships are truly due to an association between these factors, or whether hypothalamic inflammatory markers simply increased with advancing age. In future studies with larger sample sizes, correlations could be run not only by sex, but by age as well to control for the confound of age.

It is of interest to determine whether biomarkers related to metabolic function may be used to identify those at risk or in the prodromal stage of AD before the onset of cognitive decline to allow for early intervention. Longitudinal monitoring for changes in body mass would be a rapid, economical, and non-invasive option, and studies suggest that weight changes can precede cognitive symptoms by more than a decade [21–23]. It was recently reported that both weight loss and weight gain, as determined by BMI instability (> 5% increase or decrease), predicted faster cognitive decline across several domains over a 5-year period in nearly 16,000 elderly participants who were nondemented at baseline [16]. Similar findings were demonstrated in a study of 747 patients with amnestic mild cognitive impairment [18], as well as a study of 671 African American adults [17]. The latter study reported that there were no observable interactions of weight change with gender [17]. However, increased resting energy expenditure was not associated with global cognition as measured on the Mini-Mental State Examination (MMSE) or AD biomarkers [58]. We recently reported that metabolic outcomes, including weight gain, visceral adiposity, and glucose intolerance, were inversely correlated with hippocampal-dependent spatial learning and memory, as measured by performance in the Morris water maze, in both male and female 3xTg-AD mice on control or high-fat diet [62].

One limitation of the current study is that qPCR was performed to measure gene expression of markers associated with gliosis and pro-inflammatory factors. Gene expression levels do not always correlate with protein expression levels. Additionally, RNA was isolated from the dissected whole hypothalamus. Future studies using laser capture microdissection would allow for precise isolation of specific nuclei relevant to glucose and energy homeostasis (e.g., lateral hypothalamus, paraventricular nucleus, dorsomedial nucleus, ventromedial nucleus, arcuate nucleus). However, we did previously report that at ~ 7–8 months of age (approximate age at which we noted sex differences in metabolic outcomes and hypothalamic inflammatory markers shown here), both male and female 3xTg-AD mice exhibited increased Iba1 labeling in the arcuate nucleus and dorsomedial hypothalamus, in addition to increased GFAP labeling in the arcuate nucleus and dorsomedial nucleus [34]. Additionally, immunohistochemical analyses of microglia and astrocyte morphology and activation status in relevant subregions of the hypothalamus would provide additional key information. Future studies must also elucidate the precise mechanisms by which alterations in hypothalamic inflammation contribute to metabolic disturbances and how biological sex plays a role in these outcomes. One potential neuroendocrine regulator of both energy metabolism and neuroinflammation is the hypothalamic–pituitary–adrenal (HPA) axis, one of the two main arms of the stress response. Chronic stress can lead to long-term activation of the HPA axis, which is thought to contribute to the risk for both neuropsychiatric (anxiety, depression) and neurodegenerative (AD) diseases [63]. Previous work has shown that young (3–4 months) 3xTg-AD mice exhibit activation of the HPA at an age prior to significant cognitive impairment and neuropathology [64]. Given the known sex difference in HPA axis activity [65–67], it is possible that its increased activation generally observed in females may contribute to some of the findings presented here. Sex hormones influence both inflammatory and metabolic processes [68–70], which must be taken into account as aging individuals at risk for AD experience significant changes in sex hormone levels (e.g., menopause in women and steadily declining androgens in men). In addition to the known protective effects of estrogen against metabolic disease, extensive work has been done documenting the changes in energy metabolism in the brains of females during the perimenopausal transition and following menopause due to loss of estrogen [71–74]. Integrating gonadectomy or non-surgical models of menopause would increase translational value of future work in this area. Successful investigation of these phenomena may lead to the discovery of new biomarkers or targets for treatment of AD to alleviate the growing burden of this disease.

## Data Availability

The datasets used and/or analyzed during the current study are available from the corresponding authors upon request.

## Abbreviations

Aβ: Amyloid-beta
AD: Alzheimer’s disease
ARC: Arcuate nucleus
AUC: Area under the curve
BMI: Body mass index
DMH: Dorsomedial hypothalamus
GFAP: Glial fibrillary acidic protein
GTT: Glucose tolerance test
Iba1: Ionized calcium-binding adaptor molecule 1
IL-1β: Interleukin-1 beta
IL-6: Interleukin-6
LHA: Lateral hypothalamus area
PVN: Paraventricular nucleus
SEM: Standard error of the mean
TNF-α: Tumor necrosis factor alpha
VMH: Ventromedial hypothalamus
WT: Wild-type
